# C-reactive protein and post-stroke depressive symptoms

**DOI:** 10.1038/s41598-020-58478-6

**Published:** 2020-01-29

**Authors:** Katarzyna Kowalska, Paulina Pasinska, Elzbieta Klimiec-Moskal, Joanna Pera, Agnieszka Slowik, Aleksandra Klimkowicz-Mrowiec, Tomasz Dziedzic

**Affiliations:** Department of Neurology, Jagiellonian University Medical College, Krakow, Poland

**Keywords:** Depression, Stroke

## Abstract

Our study aimed to explore the association between serum C-reactive protein (CRP) and post-stroke depressive symptoms. We prospectively recruited 572 patients with ischemic stroke or transient ischemic attack in whom serum CRP level was measured within 48 h after stroke onset. Depressive symptoms were assessed at day 8 and 3 months after stroke in 405 and 306 patients, respectively. Patients with greater depressive symptoms at day 8 and patients with greater depressive symptoms 3 months after stroke had higher CRP level (median: 7.9 vs 4.3 mg/L, P < 0.01 and 6.7 vs 3.4 mg/L, P = 0.01, respectively). In the univariate analysis, CRP > 9.2 mg/L was associated with depressive symptoms at day 8 (OR: 2.06, 95%CI: 1.30–3.28, P < 0.01) and CRP > 4.3 mg/L was associated with depressive symptoms 3 months after stroke (OR: 1.79, 95%CI: 1.06–3.02, P = 0.03). In the multivariate analysis, higher CRP level was related to depressive symptoms at day 8 (OR: 2.23, 95%CI: 1.28–3.90, P < 0.01), but not depressive symptoms 3 months after stroke (OR: 1.13, 95%CI: 0.59–2.17, P = 0.71). In conclusion, higher levels of CRP are associated with greater depressive symptoms at day 8 after stroke, but their effects on depressive symptoms 3 months after stroke are less significant.

## Introduction

Mounting evidence indicates that peripheral inflammation might contribute to the pathophysiology of major depressive disorder (MDD)^1–3^. Animal studies demonstrate that systemic inflammation might interact with the mechanisms important for depression such as, neurotransmitter metabolism, glucocorticoid receptor resistance, and neuronal plasticity^4^. Clinical studies show that circulating markers of immune activation, including cytokines, chemokines, and acute-phase proteins, are observed in the blood of individuals with MDD. The most replicated findings, confirmed by several meta-analyses, pertain to raised C-reactive protein (CRP) and interleukin-6 in a subset of MDD patients^5–8^.

About 30% of patients develop depression at any time point up to 5 years after stroke^9,10^. Post-stroke depression is associated with worse functional outcome and increased mortality^11^.

In contrast to MDD, the role of systemic inflammation in the pathobiology of post-stroke depression is not well defined. Systemic inflammatory reaction accompanies ischemic stroke. This reaction includes two components: low-grade inflammation that is related to stroke risk factors and comorbidities (e.g. atherosclerosis, hypertension, diabetes mellitus, heart failure or ischemic heart disease) and acute-phase reaction triggered by brain injury and exacerbated by post-stroke infections. As a result, blood levels of interleukin-6 and CRP rise during the first few days after stroke onset^12^. Since post-stroke systemic inflammation could be a potential therapeutic target^13^, a better understanding of relationships between peripheral inflammation and depression is clinically important.

Our study aimed to explore the association between circulating CRP and post-stroke depressive symptoms.

## Methods

### Patient selection and clinical assessment

Patients recruited to this study were selected from persons who participated in the PROPOLIS study (PRospective Observational POLIsh Study on post-stroke delirium). PROPOLIS was a prospective study conducted in the Department of Neurology, University Hospital, Krakow, Poland^14^. The main aim of the PROPOLIS was to determine the frequency, risk factors and prognosis of post-stroke delirium. Participants were recruited to this study between May 2014 and March 2016.

The inclusion criteria to the current sub-study on depressive symptoms were: (1) ischemic stroke or transient ischemic attack (TIA); (2) serum CRP measurement within 48 h after stroke onset; and (3) informed patient’s consent. The exclusion criteria were: (1) the pre-stroke diagnosis of major depressive disorder (retrieved from medical records) and (2) hemorrhagic stroke.

The Bioethics Committee of Jagiellonian University approved the study’s protocol. Each patient gave informed consent. All methods were performed in accordance with approved guidelines and regulations.

The presence of depressive symptoms was assessed at day 8 ± 1 and 3 months after stroke onset using the Patient Health Questionnaire (PHQ-9)^15,16^. Previous studies showed that PHQ-9 is a valid and clinically feasible depression screening tool for stroke^17^. Score ≥10 was considered indicative of greater depressive symptoms^18,19^. The PHQ-9 was administrated face-to-face by a trained neurologist or psychologist. Before the PHQ-9 administration, aphasia was examined using clinical methods that assessed speech fluency and content, comprehension, and naming. Patients who were not able to understand questions were excluded from the study.

The Neuropsychiatric Inventory (NPI) was used to assess neuropsychiatric disturbances occurring within the 4 weeks before admission. The NPI-Q10 subscale includes 10 behavioural items: delusions, hallucinations, agitation, depression, anxiety, euphoria, apathy, disinhibition, irritability, and aberrant motor behaviour^20,21^. A score for each item (from zero to 12) is a product of severity scale (from zero to 3) and frequency scale (from zero to 4).

The Informant Questionnaire on Cognitive Decline in the Elderly (IQCODE) with a cut-off of 3.3 was used to diagnose a pre-stroke cognitive decline^22,23^. The IQCODE consists of 26 items that rate change in patients’ intellectual abilities over the past 10 years^24^.

The core features of delirium were examined using the Brief Confusion Assessment Method^25^.

National Institute of Health Stroke Scale (NIHSS) was used to assess neurological deficit on admission^26^. A score of zero means normal function and higher scores indicate greater impairment. The total score ranges from zero to 42.

### Laboratory assays

Serum CRP level was quantified via the immunoturbidometric method (Roche Diagnostics, Mannheim, Germany). The assay detection limit was 1 mg/L.

### Statistical analysis

The χ^2^ test was used to compare proportions, while the Mann–Whitney *U*-test was used to compare continuous variables between groups. Logistic regression was used to determine the predictors of functional outcome. Variables with P < 0.05 in the univariate analysis were included in a multivariate analysis. The Box-Tidwell test was used to check the linearity of the logit for the continuous independent variables in logistic regression analysis. The receiver operating characteristic curves were used to identify an optimal cut-off level of CRP that differentiates patients with greater depressive symptoms from those with lower depressive symptoms. Since CRP level could raise within 48 h after stroke onset and its level measured within 12 h might be lower than its level measured between 24–48 h^12^, two sensitivity analyses were performed. In the first, the patients in whom the CRP level was measured within 12 h after stroke were excluded. In the second analysis, only patients with CRP measurement within 24 h after stroke were included. The calculations were performed using the program STATISTICA for Windows (version 12.5, Statsoft, Poland).

## Results

**Figure 1:**
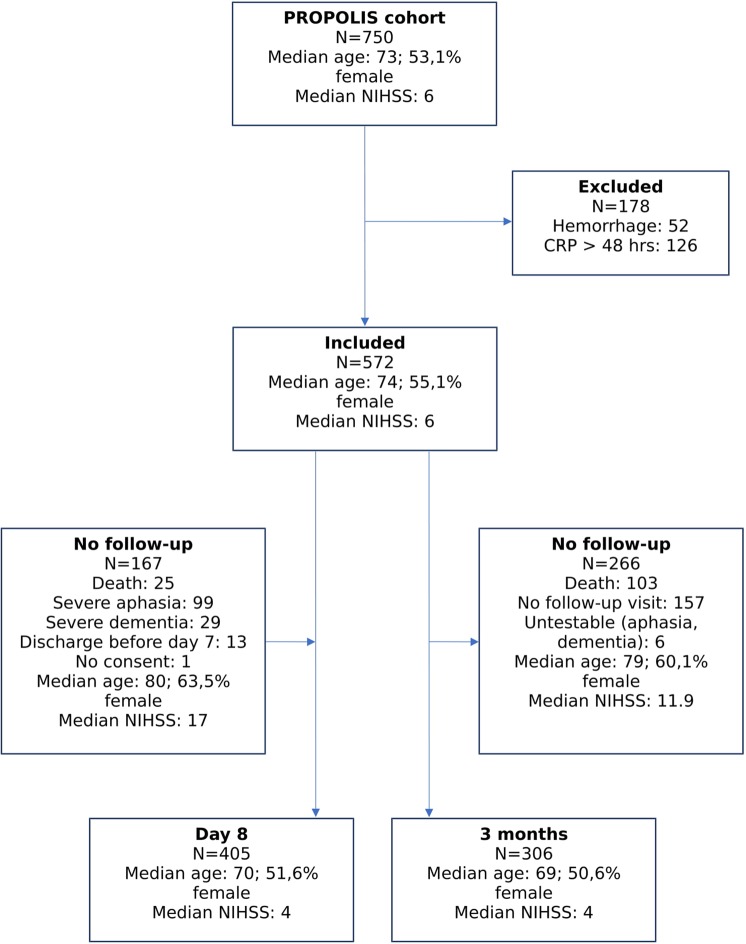
Flow chart showing the numbers of patients included in the study and the reasons for non-inclusion of excluded patients. NIHSS: National Institute of Health Stroke Scale.

Of 750 patients that participated in the PROPOLIS study, 572 patients had an ischemic stroke or TIA and the CRP measurement within 48 h after stroke onset. After the exclusion of patients who died or were not able to perform PHQ-9, depressive symptoms were assessed in 405 patients at day 8 and in 306 patients 3 months after stroke (Fig. 1).

### Depressive symptoms at day 8

Of 405 included patients (median age: 70, IQs: 61–80; 51.6% women; median NIHSS: 4, IQs: 2–9), greater depressive symptoms were diagnosed in 104 patients (25.7%).

Compared to patients with lower depressive symptoms, patients with greater depressive symptoms were more often women; suffered from hypertension, previous stroke, and pre-stroke cognitive decline; took antidepressants before index stroke; and had higher pre-stroke NPI score (Table 1). Serum CRP level was higher in patients with greater depressive symptoms.

**Table 1:** Baseline characteristics of patients with high and patients with low depressive symptoms score at day 8.

	High score of depressive symptoms (N = 104)	Low score of depressive symptoms (N = 301)	P value
Age, median (IQs)	73 (61–82)	69 (61–80)	0.20
Female, n (%)	68 (65.4)	141 (46.8)	<0.01
Hypertension, n (%)	81 (77.9)	204 (67.8)	0.05
Diabetes mellitus, n (%)	33 (31.7)	73 (24.2)	0.13
Atrial fibrillation, n (%)	24 (23.1)	48 (15.9)	0.10
Myocardial infarction, n (%)	17 (16.3)	40 (13.3)	0.44
Previous stroke or TIA, n (%)	27 (26.0)	44 (14.6)	<0.01
Pre-stroke dependency, n (%)	11 (10.6)	19 (6.3)	0.15
Pre-stroke cognitive decline, n (%)^*^	21/87 (24.1)	33/249 (13.2)	0.02
Pre-stroke total NPI score, median (IQs)**	4.5 (0–14.5)	0 (0–6)	<0.01
Pre-stroke NPI score for depression, median (IQs)**	0 (0–2)	0 (0–0)	<0.01
Pre-stroke use of antidepressants	6 (5.8)	4 (1.3)	0.01
NIHSS score on admission, n (%)	4 (2–9)	4 (2–9)	0.90
Pneumonia, n (%)	8 (7.7)	16 (5.3)	0.38
Urinary tract infections, n (%)	33 (31.7)	82 (27.2)	0.52
Delirium, n (%)	22 (21.1)	49 (16.3)	0.26
Stroke location, n (%)			0.07
Right hemisphere	53 (51.0)	129 (42.9)	
Left hemisphere	35 (33.6)	132 (43.8)	
Posterior fossa	11 (10.6)	37 (12.3)	
Multiple locations	5 (4.8)	3 (1.0)	
Intravenous thrombolysis, n (%)	22 (21.1)	82 (27.2)	0.22
Mechanical thrombectomy, n (%)	6 (5.8)	17 (5.6)	0.96
CRP (mg/L),median (IQs)	7.9 (2.4–20.5)	4.3 (2.1–10.8)	<0.01

^*^Data available for 336 patients.

**Data available for 333 patients.

In the univariate analysis, CRP level above 9.2 mg/L was associated with greater depressive symptoms (OR: 2.06, 95%CI: 1.30–3.28, P < 0.01). In the multivariate analysis adjusted for hypertension, female sex, previous stroke, pre-stroke cognitive decline, the use of anti-depressant before stroke, and the NPI score, CRP level remained the independent predictor of depression (OR: 2.23, 95%CI: 1.28–3.90, P < 0.01). Other independent predictors of greater depressive symptoms were: female sex (OR: 2.97, 95%CI: 1.67–5.29, P < 0.01), previous stroke (OR: 2.08, 95%CI: 1.08–4.01, P = 0.03), the NPI score (OR: 1.05, 95%CI: 1.02–1.08, P < 0.01) and the use of anti-depressant before stroke (OR: 6.07, 95%CI: 1.14–32.17, P = 0.03).

### Depressive symptoms 3 months after stroke onset

Of 306 included patients (median age: 69, IQs: 61–79; 50.6% women; median NIHSS: 4, IQs: 2–8), greater depressive symptoms were diagnosed in 82 patients (26.8%).

Compared to patients with lower depressive symptoms, patients with greater depressive symptoms more often suffered from hypertension, diabetes mellitus, and post-stroke delirium; took antidepressants during 3 months after stroke onset; and had higher pre-stroke NPI score (Table 2). Serum CRP level was higher in patients with greater depressive symptoms.

**Table 2:** Baseline characteristics of patients with high and patients with low depressive symptoms score at day 90.

	High score of depressive symptoms (N = 82)	Low score of depressive symptoms (N = 224)	P value
Age, median (IQs)	70.5 (63–78)	68 (60–79)	0.30
Female, n (%)	47 (57.3)	108 (48.2)	0.16
Hypertension, n (%)	68 (82.9)	150 (67.0)	0.01
Diabetes mellitus, n (%)	34 (41.5)	49 (21.9)	<0.01
Atrial fibrillation, n (%)	16 (19.5)	40 (17.9)	0.74
Myocardial infarction, n (%)	12 (14.6)	30 (13.4)	0.78
Previous stroke or TIA, n (%)	16 (19.5)	38 (17.0)	0.20
Pre-stroke dependency, n (%)	8 (9.8)	12 (5.4)	0.17
Pre-stroke cognitive decline, n (%)	13/67 (19.4)	22/191 (11.5)	0.10
Pre-stroke total NPI score, median (IQs) **	3.5 (0–12)	1 (0–7)	0.03
Pre-stroke NPI score for depression, median (IQs)**	0 (0–0)	0 (0–0)	0.94
Pre-stroke use of antidepressants	3 (3.7)	2 (0.9)	0.09
NIHSS score on admission, n (%)	5 (2–10)	4 (2–8)	0.07
Pneumonia, n (%)	8 (9.8)	12 (5.4)	0.17
Urinary tract infections, n (%)	28 (34.1)	52 (23.2)	0.06
Delirium, n (%)	22 (26.8)	27 (12.0)	<0.01
Stroke location, n (%)			0.57
Right hemisphere	39 (47.6)	83 (37.0)	
Left hemisphere	33 (40.2)	110 (49.2)	
Posterior fossa	9 (11.0)	29 (12.9)	
Multiple locations	1 (1.2)	2 (0.9)	
Intravenous thrombolysis, n (%)	26 (31.7)	59 (26.3)	0.35
Mechanical thrombectomy, n (%)	7 (8.5)	15 (6.7)	0.58
Use of anti-depressants 3 months after stroke	19 (23.2)	22 (9.8)	<0.01
CRP (mg/L),median (IQs)	6.7 (2.5–18.1)	3.4 (1.9–10.0)	0.01

^*^Data available for 258 patients.

**Data available for 255 patients.

In the univariate analysis, CRP level above 4.3 mg/L was associated with greater depressive symptoms (OR: 1.79, 95%CI: 1.06–3.02, P = 0.03). This association was nonsignificant in the multivariate analysis adjusted for hypertension, diabetes mellitus, delirium, the use of antidepressant and the NPI score (OR: 1.13, 95%CI: 0.59–2.17, P = 0.71). The independent predictors of greater depressive symptoms in this model were: hypertension (OR: 2.81, 95%CI: 1.27–6.20, P = 0.01), diabetes mellitus (OR: 2.13, 95%CI: 1.08–4.19, P = 0.03), and the use of antidepressant (OR: 4.36, 95%CI: 1.96–9.69, P < 0.01).

When patients treated with anti-depressants were excluded from the analysis, CRP was still associated with depressive symptoms in the univariate (OR: 2.00, 95%CI: 1.10–3.62, P = 0.02), but not in the multivariate analysis (OR: 1.04, 95%CI: 0.50–2.16, P = 0.92).

### Sensitivity analysis

In the first sub-analysis, we excluded patients in whom serum CRP level was measured within 12 h after stroke onset (N = 113). Compared to patients with lower depressive symptoms, patients with greater depressive symptoms at day 8 (median: 7.6, interquartiles: 2.1–25.5 mg/L vs median: 4.6, interquartiles: 1.9–11.2 mg/L, P = 0.02) and patients with greater depressive symptoms 3 months after stroke (median: 6.7, interquartiles: 2.7–19.1 mg/L vs median: 3.7, interquartiles: 1.6–11.1 mg/L, P = 0.02) had higher CRP level. In the univariate analysis, serum CRP level above 19.0 mg/L was associated with higher depressive symptoms at day 8 (OR: 2.45, 95%CI: 1.50–4.00, P < 0.01). Similarly, serum CRP level above 4.3 mg/L was related to higher depressive symptoms 3 months after stroke (OR: 2.14, 95%CI: 1.33–3.44, P < 0.01). In the multivariate analysis, higher CRP level remained an independent predictor of depressive symptoms at day 8 (OR: 2.13, 95%CI: 1.18–3.82, P = 0.01), but not depressive symptoms assessed 3 months after stroke (OR: 1.53, 95%CI: 0.86–2.73, P = 0.14).

In the second sub-analysis, we included only patients in whom CRP level was measured within 24 h after stroke onset (N = 292). Compared to patients with lower depressive symptoms, patients with greater depressive symptoms at day 8 (median: 9.2, interquartiles: 2.5–17.2 mg/L vs median: 4.3, interquartiles: 2.1–10.3 mg/L, P < 0.01) had higher CRP level. CRP level did not differ (P = 0.15) between patients who had greater depressive symptoms 3 months after stroke (median: 6.7, interquartiles: 2.5–11.2 mg/L) compared to patients who had lower depressive symptoms (median: 3.9, interquartiles: 2.1–11.0 mg/L). In the univariate analysis, serum CRP level above 9.17 mg/L was associated with higher depressive symptoms at day 8 (OR: 2.49, 95%CI: 1.45–4.29, P < 0.01) and CRP level above 6.7 mg/dL was associated with higher depressive symptoms 3 months after stroke (OR: 2.81, 95%CI: 0.99–3.31, P = 0.05). In the multivariate analysis, higher CRP level remained an independent predictor of depressive symptoms at day 8 (OR: 2.89, 95%CI: 1.47–5.69, P < 0.01), but not depressive symptoms assessed 3 months after stroke (OR: 1.21, 95%CI: 0.56–2.60, P = 0.62).

## Discussion

Our study revealed that higher levels of CRP are associated with greater depressive symptoms at day 8 after stroke, but their effects on depressive symptoms 3 months after stroke are less significant.

A few studies have examined the relationship between circulating CRP and risk of post-stroke depression. These studies yielded conflicting results. Jimenez *et al*. measured the serum CRP level in 134 patients with first-ever ischemic stroke^27^. Blood was collected at discharge (day 7 ± 2) and 1 month after stroke. About 19% of patients were diagnosed as having major depression at discharge according to DSM-IV criteria and 22% of patients had major depression 1 month after stroke. The authors did not find any association between CRP level and the risk of post-stroke depression. The study of Yang *et al*. included 226 ischemic stroke patients^28^. CRP level was measured within 24 h after stroke onset. Six months after stroke major depression was diagnosed in 30.5% of patients. In this study, serum CRP level above 0.85 mg/dL was associated with the increased risk of depression after adjusting for potential confounders. Similarly, Cheng *et al*. found that higher CRP level measured with 24 h after stroke onset predicts the increased risk of depression 1 year after stroke^29^.

The contribution of inflammatory factors to the pathogenesis of post-stroke depressive symptoms might be dependent on time after stroke. Depressive symptoms that occur very early after stroke onset could be a part of so-called sickness behaviour. Sickness behaviour is a set of behavioural and motivational changes triggered by acute infection or tissue injury and includes anhedonia, hyperalgesia, fever, anorexia, sleepiness, anxiety, and disinterest in social interactions^30,31^. Animal studies have demonstrated that systemic inflammation might induce both sickness behaviour and depressive-like symptoms^30^. Multiple symptoms, for example weight loss, anorexia, fatigue, hyperalgesia, anhedonia, anxiety, and neurocognitive symptoms are shared by both sickness behaviour and depression. Clinical studies involving cancer patients treated with interferon-alpha (INFα) shed light on cytokine-induced sickness behaviour and depressive symptoms. These studies have shown that somatic and vegetative symptoms appeared within 2 weeks of INFα therapy^32^. In contrast, mood and cognitive symptoms appeared later during INFα therapy and were more apparent in patients who developed major depression. The hypothesis that early-onset post-stroke depressive symptoms might be related to sickness behaviour is supported by the observation that stroke patients with early-onset (in-hospital) depression had higher frequency of vegetative (anxiety, loss of energy, morning depression, early awakening, weight loss) and melancholic (loss of interest, depressed mood, psychomotor retardation) symptoms compared to patients with late-onset depression^33^. Alternatively, early-onset post-stroke depression might represent a specific phenotype of depression with dominant somatic (vegetative) symptoms rather than sickness behaviour. Further studies with dimensional analyses of specific clusters of neuropsychiatric and somatic symptoms are needed to better characterize the relationship between sickness behaviour and post-stroke depressive symptoms. Differentiation of early depressive symptoms related to sickness behaviour from those predicting major depression could have therapeutic implications. In patients treated with INFα, mood and cognitive symptoms were more responsive, whereas vegetative symptoms, such as anorexia or fatigue, were less responsive to paroxetine treatment^32^.

In the univariate analysis, higher CRP was associated with greater depressive symptoms 3 months after stroke. This association was, however, non-significant after adjusting for potential confounders. Inflammatory markers decline gradually after stroke^13^. For this reason, the remote effect of inflammation on depressive symptoms could be weaker than in the acute phase of the stroke. Moreover, the persistence of depressive symptoms beyond the acute phase of stroke might require additional vulnerabilities, including unresolved inflammation, genetic predisposition, or alteration in neuronal networks responsible for mood regulation. We cannot exclude the possibility that our study had too low statistical power to detect an association between CRP and depressive symptoms occurring 3 months after stroke. It should also be noted that inflammatory markers might be selectively associated with only specific dimensions of depression. In MDD, circulating inflammatory markers are especially linked to atypical depression characterized by increased appetite and weight gain^34^.

There are several limitations to our study. First, the PROPOLIS was designed to determine frequency, predictors and clinical consequences of post-stroke delirium. Depressive symptoms were considered as a secondary end-point of the study and statistical power was not calculated a priori to check if the study was able to detect an association between CRP and depressive symptoms. Second, no formal psychiatric diagnosis of depression was made. Instead, we used a validated questionnaire to assess depressive symptoms. Third, the CRP level was measured only once. Repeated measurements of inflammatory parameters at different time points after stroke could give better insight into relationships between inflammation and depression. Fourth, about 27% of patients who were examined in acute stroke did not attend a control visit. These patients were older and had a more severe neurological deficit on admission. Fifth, about 13% of our patients took anti-depressants 3 months after stroke. CRP can interact with anti-depressive medication. In MDD patients, the elevated CRP level was associated with treatment resistance^35^.

There are also some advantages to our work. These include the prospective design of the study and assessment of pre-stroke psychiatric symptoms and cognitive decline.

Observational studies are not able to demonstrate if an association between CRP and depressive symptoms is causative. To verify this association, we need an interventional study that will examine if the anti-inflammatory treatment will reduce post-stroke depressive symptoms. From a clinical perspective, depression that occurs early after stroke (e.g., during the first 2 weeks after stroke onset) is associated with long-term poor functional outcome^36,37^. Thus, anti-inflammatory strategies attenuating systemic inflammatory reaction could have a beneficial effect on stroke outcome.

In conclusion, higher levels of serum CRP are associated with early depressive symptoms after ischemic stroke.

## Data Availability

The datasets generated during and/or analysed during the current study are available from the corresponding author on reasonable request.
